# Hepatic Expression Patterns of Inflammatory and Immune Response Genes Associated with Obesity and NASH in Morbidly Obese Patients

**DOI:** 10.1371/journal.pone.0013577

**Published:** 2010-10-22

**Authors:** Adeline Bertola, Stéphanie Bonnafous, Rodolphe Anty, Stéphanie Patouraux, Marie-Christine Saint-Paul, Antonio Iannelli, Jean Gugenheim, Jonathan Barr, José M. Mato, Yannick Le Marchand-Brustel, Albert Tran, Philippe Gual

**Affiliations:** 1 INSERM U895, Team 8 « Hepatic Complications of Obesity », Nice, France; 2 University of Nice-Sophia Antipolis, Faculty of Medicine, Nice, France; 3 Digestive Unit, Centre Hospitalier Universitaire of Nice, Nice, France; 4 Biological Unit, Centre Hospitalier Universitaire of Nice, Nice, France; 5 OWL Genomics, Bizkaia Technology Park, Bizkaia, Spain; 6 CIC bioGUNE, Centro de Investigación Biomédica en Red de Enfermedades Hepáticas y Digestivas (Ciberehd), Bizkaia Technology Park, Bizkaia, Spain; Pennington Biomedical Research Center, United States of America

## Abstract

**Background:**

Obesity modulates inflammation and activation of immune pathways which can lead to liver complications. We aimed at identifying expression patterns of inflammatory and immune response genes specifically associated with obesity and NASH in the liver of morbidly obese patients.

**Methodology/Principal Findings:**

Expression of 222 genes was evaluated by quantitative RT-PCR in the liver of morbidly obese patients with histologically normal liver (n = 6), or with severe steatosis without (n = 6) or with NASH (n = 6), and in lean controls (n = 5). Hepatic expression of 58 out of 222 inflammatory and immune response genes was upregulated in NASH patients. The most notable changes occurred in genes encoding chemokines and chemokine receptors involved in leukocyte recruitment, CD and cytokines involved in the T cell activation towards a Th1 phenotype, and immune semaphorins. This regulation seems to be specific for the liver since visceral adipose tissue expression and serum levels of MCP1, IP10, TNFα and IL6 were not modified. Importantly, 47 other genes were already upregulated in histologically normal liver (e.g. CRP, Toll-like receptor (TLR) pathway). Interestingly, serum palmitate, known to activate the TLR pathway, was increased with steatosis.

**Conclusion/Significance:**

The liver of obese patients without histological abnormalities already displayed a low-grade inflammation and could be more responsive to activators of the TLR pathway. NASH was then characterized by a specific gene signature. These findings help to identify new potential actors of the pathogenesis of NAFLD.

## Introduction

The incidence of overweight and obesity is rapidly increasing in many Western countries and is associated with the development of type 2 diabetes, hypertension and Non Alcoholic Fatty Liver Disease (NAFLD). NAFLD is one of the most common forms of chronic liver diseases [1]. Liver biopsies of patients with NAFLD show a spectrum of pathological changes ranging from simple steatosis to steatohepatitis (Non Alcoholic Steato-Hepatitis, NASH) and steatofibrosis leading, in some cases, to cirrhosis and hepatocellular carcinoma. Indeed, 20% of patients with biopsies-proven NASH may progress to cirrhosis [1].

NAFLD are frequently associated with visceral obesity, insulin resistance and metabolic syndrome [2] but predisposing and environmental factors could also be involved. Obesity modulates inflammation and activation of immune pathways which can affect hepatic lipid metabolism leading to hepatic injury, NASH and fibrosis. Adipose tissue and gut are potential players in the alteration of hepatic metabolism and inflammation [3]. Adipose tissue of obese patients is inflamed and the recruitment and activation of monocytes in the adipose tissue by chemokines such as Monocyte Chemoattractant Protein 1 (MCP1) and osteopontin are the principal effectors of this inflammation [4]–[6]. Inflammation causes adipocyte insulin resistance resulting in increased lipolysis and serum free fatty acids (FFA) flux, alteration of adipokine secretion and consequently hepatic fat accumulation and insulin resistance [7]–[9].

In NAFLD, adipose tissue substantially contributes to systemic TNFα, MCP1, IL6, and adiponectin which modify the hepatic inflammatory/immune system. We have shown that IL6-stimulated CRP and hepcidin expression in adipose tissue is associated with the elevated circulating levels of CRP and hypoferremia in morbidly obese patients independently of NASH [10]–[12]. Patients with NAFLD also seem to have an increased intestinal permeability associated with loss of integrity of epithelial tight junctions which could lead to increased levels of circulating lipopolysaccharide (LPS) [13]. Activation of cells including Kupffer cells by LPS triggers the production of reactive oxygen species and pro-inflammatory cytokines in the liver, resulting in NAFLD [14].

Alteration in Natural Killer T (NKT) cells number and/or activity and activation of the adaptive immune system could also promote the liver alterations. NKT cells can produce both T helper 1 (Th1) and T helper 2 (Th2) associated cytokines steering the immune system into either inflammation or tolerance. An inverse correlation has been reported between the severity of steatosis and NKT cell number in mice and the reduction in the NKT cell number was associated with an increased hepatic production of Th1 cytokines [3]. In NAFLD patients, peripheral NKT cell number is decreased [15]. Activation of Kupffer cells, oxidative stress, reduction in regulatory T cells, and the altered profiles of adipokines (leptin, resistin versus adiponectin) could also lead to the disruption of the physiological tolerance of the liver to portal antigens [3]. Indeed, IgG antibodies against lipid peroxidation-derived antigens have been observed in patients with NAFLD and are associated with advanced fibrosis [16].

The inflammation and immune reactions in the progression of normal liver to steatosis, and then NASH are not well characterized. In this paper, we have focused on the differential expression of genes related to inflammation and immune response specifically associated with obesity, steatosis and NASH in liver of morbidly obese patients. To this aim, a quantitative approach was used focussing at most inflammatory pathways reported in the hepatic complications induced by obesity and chronic alcohol consumption in mice and humans.

## Results

### Clinical and biochemical data

Characteristics of the morbidly obese patients are given in Table 1, according to the severity of steatosis (S0: without steatosis, n = 6; S3: with severe steatosis, n = 6) and the presence of NASH (NASH, n = 6) determined on liver biopsies. All NASH patients have severe steatosis and a NAFLD activity score (NAS) ≥5. All the obese patients have the same hepatic fibrosis stage (F1). A significant difference was observed in the genders of NASH obese patients and the S0 and S3 obese patients.

**Table 1:** Characteristics of the morbidly obese patients.

	S0	S3	NASH
Sex (female/male)	5/1	5/1	1/5
Age (years)	36.2±5.9	36.0±3.4	43.2±4.0
BMI (kg/m^2^)	43.8±0.6	44.3±1.9	40.2±1.3*
ALT (IU/L)	17.5±3.9	35.1±4.2*	58.0±9.7* ^/^ §
AST (IU/L)	20.0±3.8	24.9±2.2*	35.2±4.4* ^/^ §
GGT (IU/L)	19.5±2.6	33.7±7.3*	57.2±15.2*
Alkaline phosphatase (IU/L)	74.7±8.9	87.3±10.6	75.0±3.4
Albumin (g/L)	37.9±1.0	42.1±1.5*	43.9±1.3*
Total bilirubin (µmol/L)	8.8±2.4	7.1±0.5	10.4±1.7§
Conjugated bilirubin (µmol/L)	1.8±0.7	2.1±0.1	2.6±0.2
Fasting insulin (mIU/L)	7.1±0.9	13.7±3.5	26.0±6.3*
Fasting glucose (mmol/L)	4.9±0.1	5.4±0.3	7.3±1.3*
HOMA-IR	1.6±0.2	3.5±1.1*	7.9±1.7* ^/^ §
Triglycerides (mmol/L)	0.99±0.14	1.61±0.27	4.44±1.44* ^/^ §
Free fatty acids (mmol/L)	0.44±0.06	0.64±0.05	0.47±0.08
Total cholesterol (mmol/L)	4.74±0.42	5.89±0.45	6.34±0.42*
HDL cholesterol (mmol/L)	1.40±0.11	1.50±0.14	1.14±0.07
LDL cholesterol (mmol/L)	2.90±0.44	3.66±0.34	3.18±0.27
***NAFLD Activity Score (n)***	***0 (6)***	***3 (6)***	***5 (5) 6 (1)***
Grade of steatosis (n)	0 (6)	3 (6)	3 (6)
Lobular inflammation (n)	0 (6)	0 (6)	1 (5) 2 (1)
Hepatocellular ballooning (n)	0 (6)	0 (6)	1 (6)
Fibrosis (n)	1 (6)	1 (6)	1 (6)

S0: patients with normal liver histology, S3: patients with severe steatosis, NASH: patients with severe steatosis and NASH. Data are expressed as mean±SEM and were compared by using the non parametric Kruskal-Wallis test.

*P<0.05 compared with S0 patients and.

§ P<0.05 compared with S3 patients.

### Genes upregulated with NASH

The goal of this study was to better characterize the inflammation and immune reaction in NAFLD patients. To this aim, we examined the expression levels of 222 selected genes related to inflammation and immune response including the CD, chemokines, semaphorins/plexins, interleukin, IFN, TNFα, TGFβ, NFκB and Toll-like receptor pathways (Table S1) by a quantitative RT-PCR approach. Among the 222 inflammatory and immune response genes tested, 192 were detected in liver in our experimental conditions (Table S1).

**Figure 1:**
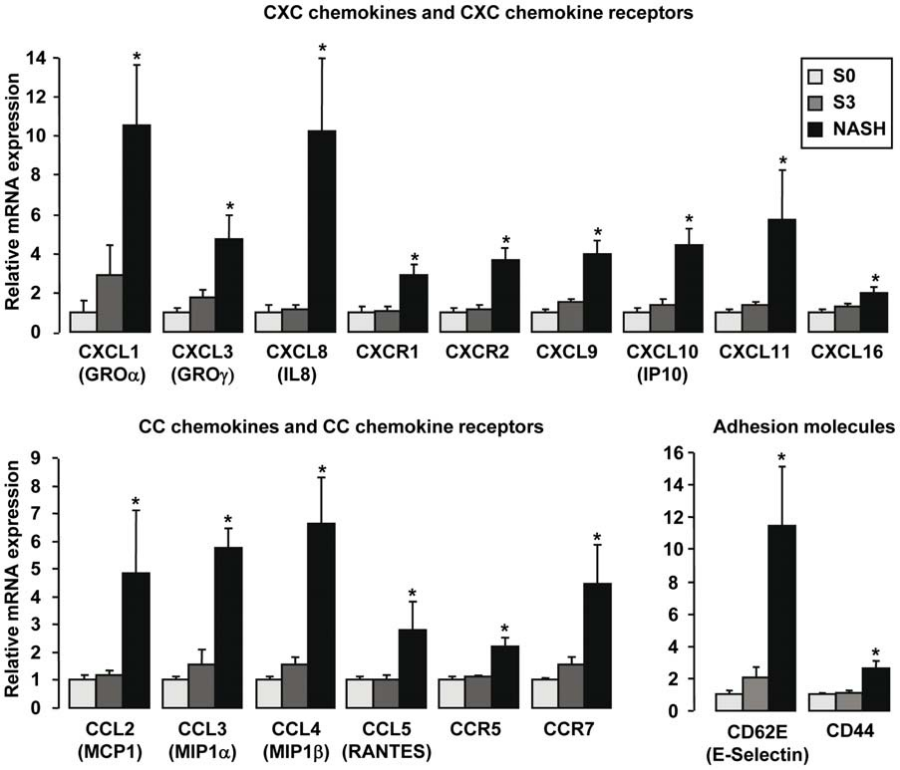
Upregulation of 17 genes encoding proteins involved in leukocyte recruitment in liver of NASH patients. The hepatic expression levels of genes were analyzed by real-time quantitative PCR in morbidly obese patients with normal liver histology (S0, n = 6), in morbidly obese patients with severe steatosis (S3, n = 6), and in morbidly obese patients with severe steatosis and NASH (NASH, n = 6). The mRNA levels of genes were normalized to the mRNA levels of RPLP0. Results are expressed relative to the expression levels in S0 patients and expressed as mean±SEM. *P<0.05, compared with S0 patients.

**Figure 2:**
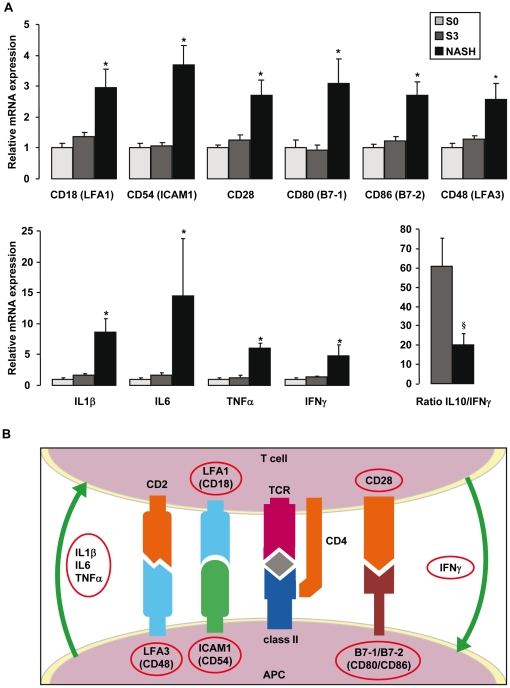
Specific upregulation of genes encoding Th1 cytokines and proteins involved in recognition between APC and T cells in liver of NASH patients. **A.** The hepatic expression levels of genes were analyzed by real-time quantitative PCR in morbidly obese patients with normal liver histology (S0, n = 6), in morbidly obese patients with severe steatosis (S3, n = 6), and in morbidly obese patients with severe steatosis and NASH (NASH, n = 6). The mRNA levels of genes were normalized to the mRNA levels of RPLP0. Results are expressed relative to the expression levels in S0 patients and expressed as mean±SEM. *P<0.05, compared with S0patients; ^§^P = 0,033. **B.** Scheme of recognition between APC and T cells.

A number of comparisons were then performed to detect genes potentially involved in obesity, liver steatosis and NASH. The first analysis compared expression profiles between NASH and S3 patients. We defined as a NASH specific gene, a gene which was upregulated more than 1.4 fold in NASH patients compared to S3 patients and, evidently, to S0 patients and controls (lean patients without liver disease). 58 genes were upregulated in NASH patients and can be separated into two groups: 38 genes were specifically upregulated in NASH patients (and not in S0 and S3 patients) and 20 genes were upregulated in S0 and S3 patients and further increased in NASH patients (Table 2). Interestingly, as illustrated in Figure 1, 15 genes upregulated in NASH patients encoded chemokines and chemokine receptors involved in leukocyte recruitment including the couples CXCL8/CXCR1; CXCL1, 3/CXCR2; CCL3-5/CCR5 and the chemokines CXCL9-11 and CCL2 (MCP1). In addition, CD62E (E-Selectin) and CD44 which could be involved in leukocyte recruitment into inflammation sites were strongly upregulated in NASH patients (Figure 1). Some of the NASH specific genes encoded cytokines and molecules involved in the interaction and co-stimulation between antigen-presenting cells (APC) and T lymphocytes (Figure 2) leading to T cell activation towards a Th1 phenotype. Indeed, the gene expression levels of CD28, CD80 (B7-1), CD86 (B7-2) and IFNγ, in addition to CD18 (LFA1), CD54 (ICAM1), IL1β, IL6 and TNFα were upregulated only in NASH patients. Moreover, the ratio IL10 versus IFNγ was strongly decreased in NASH patients compared to S3 patients (Figure 2A). Among the other NASH genes, the expression of genes encoding members of the plexin/semaphorin family (PLXNC1; SEMA 4D, 7A and 4A) was also strongly increased in NASH patients (Table 2). We finally confirmed the modification of the expression levels of TNFα, IL6 and CCL2 (MCP1) in NASH patients previously reported by other groups [17]–[20].

**Table 2:** List of the 58 genes specifically upregulated in liver of NASH patients.

Gene symbol	Controls	S0	S3	NASH	Fold NASH vs S3	*P* NASH vs S3
***CD***						
CD62E/E-Selectin	1.00±0.35	4.39±1.37*	9.19±2.52*	50.28±15.99*	5.5	0.005
CD69/EA1	1.00±0.16	1.63±0.25	1.76±0.47	6.22±1.78*	3.5	0.016
CD54/ICAM1	1.00±0.16	1.76±0.24*	1.88±0.21*	6.48±1.10*	3.5	0.006
CD80/B7-1	1.00±0.06	2.79±0.70*	2.59±0.49*	8.62±2.22*	3.3	0.025
CD11b/ITGAM	1.00±0.18	1.20±0.13	1.38±0.20	4.15 ±1.30*	3.0	0.006
CD44	1.00±0.18	1.77±0.19*	2.03±0.16*	4.63±0.89*	2.3	0.006
CD18/LFA1	1.00±0.21	0.97±0.14	1.31±0.14	2.85±0.59*	2.2	0.010
CD86/B7-2	1.00±0.25	1.79±0.23	2.20±0.26	4.83±0.79*	2.2	0.008
CD28	1.00±0.15	1.33±0.12	1.69±0.21	3.57±0.67*	2.1	0.046
CD48/LFA3	1.00±0.27	1.13±0.17	1.45±0.12	2.90±0.59*	2.0	0.010
CD68	1.00±0.10	2.20±0.19*	2.25±0.20*	4.12±0.42*	1.8	0.010
***Chemokines and chemokine receptors***						
CXCL8/IL8	1.00±0.10	7.21±2.79*	8.12±1.76*	73.71±27.14*	9.1	0.006
CCL4/MIP1b	1.00±0.15	1.17±0.18	1.81±0.34	7.78±1.95*	4.3	0.006
CXCL11	1.00±0.23	1.25±0.16	1.72±0.19	7.19±3.08*	4.2	0.010
CCL2/MCP1	1.00±0.32	2.58±0.48*	3.08±0.32*	12.52±5.78*	4.1	0.037
CXCL1/GROα	1.00±0.49	2.46±1.42	7.13±3.72	26.03±7.49*	3.7	0.016
CCL3/MIP1a	1.00±0.19	2.45±0.35*	3.80±1.39*	14.19±1.64*	3.7	0.011
CXCL10/IP10	1.00±0.32	0.81±0.20	1.11±0.27	3.58±0.66*	3.2	0.010
CXCR2/IL8RB	1.00±0.21	1.66±0.42	1.93±0.30	6.12±0.97*	3.2	0.006
CCR7	1.00±0.15	1.48±0.09*	2.28±0.40*	6.63±2.06*	2.9	0.045
CXCL3/GROγ	1.00±0.36	2.91±0.76*	5.02±1.21*	13.82±3.59*	2.8	0.028
CXCL9	1.00±0.29	0.74±0.12	1.10±0.12	2.91±0.52*	2.7	0.016
CCL5/RANTES	1.00±0.21	0.88±0.12	0.92±0.10	2.48±0.89*	2.7	0.037
CXCR1/IL8RA	1.00±0.18	2.54±0.86*	2.77±0.52*	7.39±1.31*	2.7	0.010
CCR5	1.00±0.14	0.84±0.11	0.94±0.03	1.87±0.24*	2.0	0.004
CXCL16	1.00±0.11	1.19±0.18	1.55±0.17	2.38±0.39*	1.5	0.025
***Semaphorins and plexins***						
PLXNC1	1.00± 0.07	1.35±0.23	1.40±0.12	5.70±2.28*	4.1	0.010
SEMA4A	1.00± 0.20	1.55± 0.42	1.48±0.24	5.36±1.66*	3.6	0.016
SEMA7A	1.00±0.25	0.87±0.09	0.97±0.08	2.87±0.71*	3.0	0.010
SEMA4D	1.00±0.19	1.25±0.13	1.42±0.10	3.43±1.22*	2.4	0.037
***Interleukin pathway***						
IL6	1.00±0.42	4.01±0.96	3.58±1.67	21.27±6.78*	5.9	0.011
IL1B	1.00± 0.37	0.90±0.17	1.41±0.27	7.68±2.04*	5.4	0.003
IL7R	1.00±0.23	1.39±0.15	1.29±0.12	4.40±1.56*	3.4	0.028
IL1RN	1.00±0.10	1.61±0.25*	1.78±0.32*	5.15±0.77*	2.9	0.010
IL3RA	1.00±0.23	1.06±0.25	1.26±0.11	3.32±1.12*	2.6	0.016
NFL3	1.00±0.11	1.62± 0.20*	1.86±0.24*	4.90±0.80*	2.6	0.004
IL2RG	1.00±0.16	0.91±0.09	1.01±0.12	2.56±0.53*	2.5	0.016
IL18	1.00±0.33	1.08±0.14	1.16±0.12	2.44±0.54*	2.1	0.010
IL13RA2	1.00±0.18	1.02±0.14	0.89±0.18	1.86±0.32*	2.1	0.013
IL27RA	1.00±0.17	0.70±0.09	1.00±0.15	1.93±0.34*	1.9	0.028
IL1R2	1.00±0.14	1.27±0.24	1.23±0.17	2.02±0.17*	1.6	0.018
IL18BP	1.00±0.06	1.47±0.15	2.00±0.22	3.07±0.27*	1.5	0.013
***JAK-STAT-SOCS pathway***						
SOCS3	1.00±0.34	3.63±0.73*	3.64±0.81*	13.37±3.34*	3.7	0.028
SOCS1	1.00±0.11	2.06±0.44*	1.78±0.23*	5.10±1.44*	2.9	0.025
***IFN pathway***						
IFNG	1.00±0.28	0.86±0.20	1.13±0.21	4.12±1.46*	3.6	0.039
IFI16	1.00±0.25	1.42±0.17	1.20±0.09	3.35±1.02*	2.4	0.008
***TNFα and TGFβ pathways***						
TNF	1.00±0.37	0.99±0.22	1.28±0.30	5.90±0.91*	4.6	0.004
LTB	1.00±0.20	0.90±0.15	1.15±0.14	3.28±1.05*	2.9	0.019
TNFRSF1B	1.00±0.12	1.54±0.10	1.44±0.17	3.40±0.85*	2.4	0.016
TRAF1	1.00±0.12	1.06±0.14	1.24±0.17	2.70±0.43*	2.2	0.019
TGFB1	1.00±0.14	1.27±0.22	1.54±0.13	2.70±0.51*	1.8	0.016
***NFκB pathway***						
REL	1.00±0.22	1.72±0.23*	2.18±0.29*	5.12±1.28*	2.4	0.014
RELB	1.00±0.24	2.53±0.49*	2.37±0.33*	5.40±0.84*	2.3	0.010
NFKB2	1.00±0.18	1.77±0.26*	1.86±0.17*	3.22±0.46*	1.7	0.037
***Matrix proteases and inhibitors of matrix proteases***						
MMP9	1.00±0.26	2.35±0.41*	3.63±0.68*	16.54±4.00*	4.6	0.011
SERPINE1/PAI1	1.00±0.35	1.95±0.83*	5.65±0.75*	25.39±4.18*	4.5	0.004
TIMP1	1.00±0.12	0.94±0.19	1.04±0.15	2.98±0.86*	2.9	0.016
PLAU	1.00±0.18	1.25±0.30	1.76±0.27	4.79±0.80*	2.7	0.006

S0: patients with normal liver histology (n = 6), S3: patients with severe steatosis (n = 6), NASH: patients with severe steatosis and NASH (n = 6). Results are expressed relative to control subjects (mean±SEM) and were compared by using the non parametric Kruskal-Wallis test.

*P<0.05 compared with controls.

**Figure 3:**
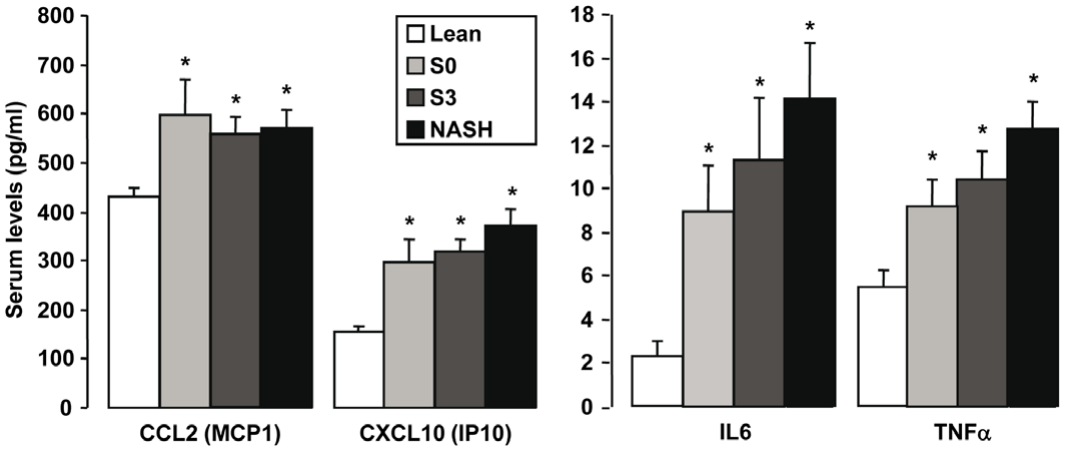
Elevated serum levels of CCL2, IP10, IL6 and TNFα are dependent on obesity but not on liver complications. The serum of 9 lean patients and 70 morbidly obese patients (15 S0 patients, 23 S3 patients and 23 NASH patients with severe steatosis) were used to evaluate the circulating levels of CCL2, IP10, IL6 and TNFα.

Since inflamed adipose tissue could contribute to liver complications, we then evaluated in visceral adipose tissue (VAT) the gene expression levels of the NASH markers identified in the liver. VAT was chosen for these studies since adipokines secreted by VAT are directly delivered to the liver via the portal vein. Only 10 genes out of the 58 NASH specific genes had a differential expression in the VAT of NASH patients versus S3 and S0 patients (Table S2). This indicates that the majority of the NASH markers identified are liver specific. We then evaluated the circulating levels of two chemokines (MCP1, IP10) and two cytokines (IL6, TNFα) which are strongly upregulated in the liver but not in the adipose tissue of NASH patients, in a larger number of patients (10 lean, 17 S0, 24 S3 and 27 NASH patients). As shown in Figure 3, the expression level of these chemokines and cytokines was similarly increased in all obese patients compared with lean subjects with no further increase in NASH patients. This result indicates that the adipose tissue could be the main source of these circulating factors but that their local hepatic upregulation could play an important role in the evolution of the liver complications.

### Genes specifically upregulated with severe steatosis

We then compared expression of genes in severe steatotic livers (S3 obese patients) with livers without any histological alterations (S0 obese patients). Surprisingly, we identified only 3 genes which were upregulated. They encoded SEMA3C (3.6 fold increase, P = 0.001), IL10 (3.3 fold increase, P = 0.005) and CLDN10 (2.2 fold increase, P = 0.002).

### Genes differentially expressed in liver of S0 obese patients compared with lean controls

**Figure 4:**
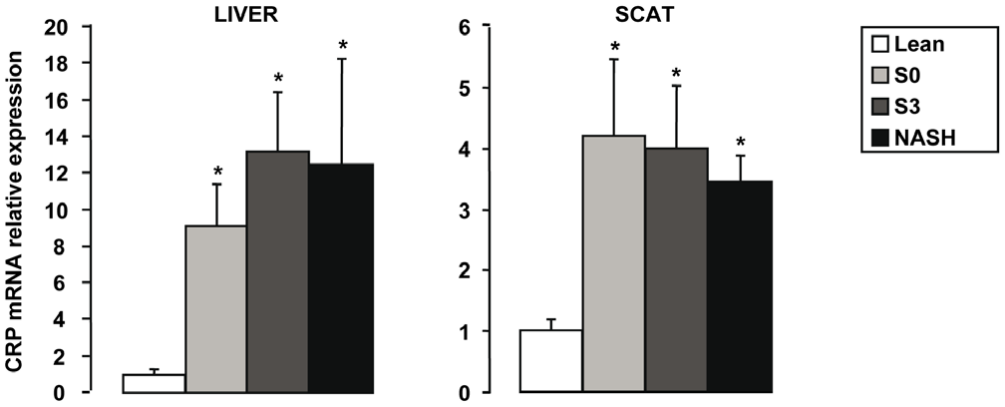
CRP gene expression levels in function to liver complications. The expression levels of CRP were analyzed by real-time quantitative PCR in lean subjects (n = 6 for the liver; n = 4 for the SCAT), in morbidly obese patients with normal liver histology (S0,n = 6), in morbidly obese patients with severe steatosis (S3, n = 6), and in morbidly obese patients with severe steatosis and NASH (NASH, n = 6) in liver and subcutaneous adipose tissue. The mRNA levels of genes were normalized to the mRNA levels of RPLP0. Results are expressed relative to the expression levels in controls and expressed as mean±SEM. *P<0.05, compared with controls.

**Figure 5:**
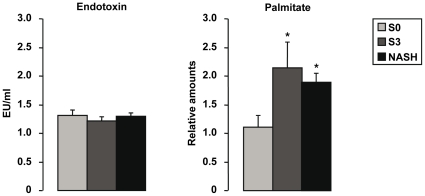
Elevated serum palmitate levels but not endoxin levels were present in severely steatotic obese patients. **A.** The serum of 9 lean patients and 70 morbidly obese patients (15 S0 patients, 23 S3 patients and 23 NASH patients with severe steatosis) was used to evaluate the circulating levels of endotoxin. **B.** The abundances of palmitate as expressed relative to their values in a commercial serum sample (over 1000 individuals) were evaluated in 8 S0 patients, 9 S3 patients and 8 NASH patients.

Differential expression between lean controls and obese patients without histological features of steatosis and inflammation (S0 obese patients) was evaluated to identify genes which could be related mostly to obesity. We have previously reported in the serum, liver and adipose tissue of morbidly obese patients that elevated CRP expression levels were independent of metabolic syndrome, type 2 diabetes and NASH [10]. We further show here that CRP gene expression in liver and subcutaneous adipose tissue was upregulated in all morbidly obese patients independently of liver complications (Figure 4). This later study was performed only in SCAT since control VAT was not available. Furthermore, we identified 46 other genes upregulated in the liver of all obese patients (Table 3) with no difference between S0, S3 and NASH patients (data not shown). Interestingly, the expression levels of gene encoding proteins involved in Toll-like receptors (TLR) and LPS signaling pathways including CD14, TLR4, TLR6, TLR2, TRAF3, TRAF6 and CHUK (IKKα) were upregulated in obese patients compared with controls (Table 3). CD14/TLR4 and TLR2/TLR6 are receptors for LPS and diacyl lipopeptides, respectively [14]. Saturated fatty acids are also known to stimulate TLR4 in macrophages [21]. We have thus evaluated the circulating levels of LPS, total free fatty acids and palmitate in the serum of some obese patients without or with hepatic histological alterations. While the endotoxemia (Figure 5) and total free fatty acids (Table 1) were not modified, the relative expression of palmitate (Figure 5) was increased in patients with severe steatosis or NASH compared to morbidly obese patients without liver alterations.

**Table 3:** List of the 47 genes specifically upregulated in liver of obese patients.

Gene symbol	Controls (n = 5)	Obese (n = 18)	*P*
***Acute phase protein***			
CRP	1.00±0.33	11.71±2.20	0.003
***Toll-like receptors and lipopolysaccharide pathway***			
TLR2	1.00±0.17	4.83±0.83	0.003
TLR6	1.00±0.21	3.09±0.32	0.002
TLR4	1.00±0.13	2.87±0.34	0.002
CD14	1.00±0.10	1.88±0.14	0.002
TRAF6	1.00±0.10	1.78±0.11	0.002
CHUK/IKKα	1.00±0.12	1.77±0.13	0.003
TRAF3	1.00±0.07	1.51±0.09	0.007
***CD***			
CD180	1.00±0.07	2.89±0.18	0.002
CD38	1.00±0.15	2.81±0.26	0.007
CD36	1.00±0.12	2.69±0.32	0.001
CD4	1.00±0.17	2.31±0.19	0.003
CD62P/P-Selectin	1.00±0.11	1.90±0.25	0.005
CD3E	1.00±0.10	1.87±0.17	0.011
CD40	1.00±0.14	1.78±0.10	0.002
CD47	1.00±0.07	1.56±0.09	0.001
***Chemokines and chemokine receptors***			
CXCR4	1.00±0.13	4.05±0.65	0.003
CXCL2	1.00±0.29	2.43±0.26	0.009
***Semaphorins, plexins and neuropilins***			
PLXNA2	1.00±0.12	2.55±0.24	0.001
SEMA4F	1.00±0.10	2.41±0.16	0.002
SEMA5A	1.00±0.11	2.40±0.16	0.001
NRP2	1.00±0.15	2.35±0.19	0.003
SEMA5B	1.00±0.11	1.99±0.20	0.002
SEMA6B	1.00±0.09	0.32±0.04	0.001
***Interleukin pathway***			
IL2RA	1.00±0.32	7.57±1.71	0.005
IL12A	1.00±0.31	2.60±0.35	0.013
IL16	1.00±0.13	2.52±0.19	0.001
IL1R1	1.00±0.13	2.20±0.16	0.001
IL22RA1	1.00±0.09	2.13±0.20	0.003
IL18R1	1.00±0.19	2.09±0.21	0.013
IL10RA	1.00±0.14	1.92±0.19	0.004
IL17R	1.00±0.07	1.88±0.17	0.002
IL4R	1.00±0.15	1.75±0.14	0.006
***JAK-STAT-SOCS pathway***			
STAT2	1.00±0.14	1.89±0.16	0.006
SOCS7	1.00±0.05	1.82±0.11	0.003
JAK1	1.00±0.11	1.71±0.10	0.002
***TNF*** **α ** ***and TGFβ pathways***			
TRAF5	1.00±0.12	2.27±0.23	0.003
TGFBR1	1.00±0.14	1.83±0.13	0.007
TNFRSF14	1.00±0.08	1.48±0.08	0.007
***IFN pathway***			
IFNGR1	1.00±0.09	2.86±0.33	0.001
IRF6	1.00±0.11	2.04±0.13	0.003
IRF2	1.00±0.07	2.01±0.13	0.002
IFNAR1	1.00±0.17	1.79±0.10	0.005
IFNAR2	1.00±0.04	1.76±0.08	0.001
***NFkB pathway***			
NFKBIA	1.00±0.09	2.90±0.36	0.001
***c-Jun N-terminal kinases***			
MAPK8/JNK1	1.00±0.08	2.35±0.13	0.001
***Matrix proteases***			
MMP14	1.00±0.14	2.09±0.17	0.002

Results are expressed relative to control subjects (mean±SEM) and were compared by using the non parametric Kruskal-Wallis test.

## Discussion

We report here that the hepatic expression levels of several actors of the inflammatory and immune responses are modified in all morbidly obese patients even without histological features of steatosis or inflammation. Furthermore, the liver of obese patients could be more responsive to activators of the TLR pathway (endotoxin, saturated fatty acids) compared to lean subjects. The NASH status appears preferentially associated with the cell recruitment mediated by chemokines, a better recognition between APC and T cells and a Th1 response steering the immune system into inflammation.

Since a significant difference in the genders between the NASH obese patients (predominantly male) and the S0 and S3 obese patients (predominantly female) was present in our study, this should be taken into account in the interpretation of the results because a gender specific gene expression in the liver has been reported [22], [23]. However, the prevalence of NAFLD is significantly different between genders and was higher in men that in premenopausal women [24]. Clinical evidence in other chronic liver diseases (i.e. chronic viral hepatitis) and evidence in experimental models suggest that estrogen may be protective against the NAFLD progression [24]. Nevertheless, a gender effect can be ruled out in our studies since the same expression pattern of hepatic genes upregulated in obesity was found in the S0, S3 and NASH obese patients when compared with lean control subjects.

We first identified fifty eight genes upregulated in NASH patients. TNFα, IL6, IL1β, CXCL8 and TGFβ, which are obvious actors in the pathogenesis of progressive NAFLD, are involved in hepatocytes death/apoptosis (TNFα, TGFβ), neutrophil chemotaxis (CXCL8), activation of hepatic cells (TNFα, TGFβ), Mallory-Denk bodies (TNFα, TGFβ) and hepatic insulin resistance (TNFα, IL6, IL1β and SOCS3) [25]. This is in agreement with the increased hepatic expression of IL6, TNFα and TNF receptors which has been correlated with histological severity in obese patients [17], [18].

Several studies have suggested that the homing of circulating lymphocytes to the liver may increase during inflammation and enhance hepatic inflammation [26]. A recent study has demonstrated that antibiotic treatment of *ob/ob* mice decreased hepatic infiltration of CD4^+^ T, CD8^+^ T, NKT, B and NK cells [27]. In our patients, we show here that gene expression of CXCL8 (IL8) and its receptor CXCR1; CXCL1, 3 and their receptor CXCR2; CCL3-5 and their receptor CCR5; CCL2 (MCP1); CXCL9-11; CD62E (E-Selectin) and CD44 are strongly upregulated in NASH patients. The chemokine expression thus appears to be more related to inflammation and hepatocellular ballooning than to severe steatosis. While the hepatic upregulation of these actors could be important in the progression of liver complications, the real contribution of these proteins for inflammatory cells recruitment into the liver has to be determined, since systemic levels of IL6, TNFα, IP10 and MCP1 are dependent on obesity. The highest upregulated chemokine in liver is CXCL8 (IL8) (73 fold increases compared to the lean controls). The non parenchymal cells but also hepatocytes should contribute to its expression. Indeed, it has been recently reported that palmitate induces production of IL8 in hepatocytes [28].

Although NASH is not classically considered a Th1-polarized disease, our data suggest that an imbalance resulting from a relative excess in pro-inflammatory Th1 cytokines such as IFNγ, and a relative deficiency of anti-inflammatory cytokines such as IL10 is associated with NASH. Expression of genes encoding proteins required for the interaction and co-stimulation between APC and T cells (CD28, CD80, CD86, CD18, CD54) is also specifically upregulated in NASH patients. It has been recently shown that the serum CD54 (ICAM1) concentration is increased in patients with NASH [29]. In addition, we have previously reported the potential role of hepatic osteopontin, a Th1 cytokine, in human NAFLD [6]. Osteopontin could enhance the activation of the Th1 immune reaction and decrease the expression of IL10 [30]–[32]. In mice models of NAFLD, hepatic NKT cells and the Th1 immune reaction have been recently implicated in the pathogenesis of NASH. Steatohepatitis is associated with a reduction in the number of hepatic NKT cells, a high sensitivity to the endotoxin-induced Th1 cytokine production [33], an impairment of the Kupffer cells functions [34], a reduction in the serum levels of IL10 and 15 and an increase in the IL12 levels [34]–[36] and excessive hepatic production of Th1 cytokines promoting inflammation [37]. Oral immunization against liver-extracted proteins was associated with a shift from a Th1 to Th2 immune reaction, leading to the amelioration of NASH and glucose intolerance in the leptin-deficient mouse model [38]. The peripheral NKT cells number is also decreased in patients with NAFLD [15] and an elevated Th1-cytokine profile dominated by the production of IFNγ is correlated with insulin resistance and NASH in obese children [39]. While additional investigations are required, all these reports and our results are in favor of a role of Th1 reaction in the pathogenesis of NASH.

We also show, for the first time, that a large number of plexin/semaphorin family members (28 members) are expressed in the human liver and that the expression of the “immune semaphorins” (SEMA4A, SEMA7A, SEMA4D, and PLXNC1) is upregulated in NASH patients. SEMA4D is crucially involved in the activation and differentiation of T cells, SEMA4A may promote Th1 differentiation and SEMA7A stimulates macrophages to produce pro-inflammatory cytokines [40], [41].

In this study, we have validated the hepatic specificity of our NASH markers since the same genes were not modified in VAT. However, specific modifications of the VAT inflammation have been correlated with the evolution of liver complications. For example, increased infiltration of macrophages in omental adipose tissue was associated with marked hepatic lesions in morbid human obesity [42], [43]. Prominent adipose specific deregulation of inflammation and immune system related genes has been also recently reported in morbidly obese patients in function to NASH, fibrosis and type 2 diabetes [44], [45]. These differences could be due to the presence of type 2 diabetes and above all the stage of hepatic fibrosis. All our patients have the same hepatic fibrosis stage.

Surprisingly, we observed the presence of low-grade inflammation in the liver of morbidly obese patients without any obvious histopathological abnormalities (S0 patients) as indicated by the upregulation of 47 genes related to inflammation and immune response. For example, the hepatic expression of CRP, a non-specific marker of inflammation, was elevated in all obese patients in accordance with the moderate elevation of CRP plasma levels in the cohort of severely obese patients [10]. The increased circulating IL6, mainly due to adipose tissue secretion, could enhance the hepatic expression of CRP. The upregulation of key molecules required for the TLR signaling pathways (CD14, TLR4) were also suggestive of a higher responsiveness to LPS or saturated fatty acids in obese patients. A series of reports are in favor of a role of LPS, mainly through TLR4 and CD14, in obesity induced NAFLD [46]–[52]. In fasted serum from our obese patients, the endotoxin level was similar in all groups of patients. This observation should however be taken with caution since it could be different upon meal challenge. In contrast, the relative amount of palmitate was increased in steatotic patients. It is now known that the saturated fatty acids can activate macrophages via TLR4 [21]. This hepatic inflammation could be involved in the development of liver complications since activation of Kupffer cells was a causal factor for hepatic steatosis and insulin resistance [53], [54].

In summary, the liver of morbidly obese patients without histological abnormalities had a low-grade inflammation characterised by the elevated expression of inflammatory markers and could be more responsive to endotoxin and saturated fatty acids due to the elevated expression of the Toll-like receptors. The transient and repeated activation of the TLR pathway by the transient upregulation of activators of the TLR could be involved in the evolution of liver complications. Furthermore, the studies focusing on the behavior of identified chemokines, semaphorins and the actors leading to Th1 response on the liver functions are attractive approaches to acquire more insight into the pathogenesis of human NAFLD.

## Materials and Methods

### Study population

#### Patient population

18 morbidly obese patients were recruited through the Department of Digestive Surgery and Liver Transplantation where they underwent bariatric surgery for their morbid obesity (Nice hospital). Bariatric surgery was indicated for these patients in accordance with the French Guidelines. Exclusion criteria were: presence of hepatitis B or hepatitis C virus infection, excessive alcohol consumption (>20 g/d) or another cause of chronic liver diseases as previously described [6], [10], [11]. The characteristics of the study groups are described in Table 1. Before surgery, fasting blood samples were obtained and used to measure alanine amino transferase (ALT), aspartate aminotransferase (AST), gamma glutamyl transferase (GGT), alkaline phosphatase, albumin, total and conjugated bilirubin. Insulin resistance was calculated by using the homeostatic model assessment (HOMA-IR) index [55]. Surgical liver biopsies were obtained during surgery. Histopathological analysis was performed according to the scoring system published by Kleiner and *al*. [56]. Four histopathological features were semi-quantitatively evaluated: grade of steatosis (0, <5%; 1, 5%–30%; 2, >30%–60%; 3, >60%), lobular inflammation (0, no inflammatory foci; 1, <2 inflammatory foci per 200x field; 2, 2–4 inflammatory foci per 200x field; 3, >4 inflammatory foci per 200x field), hepatocellular ballooning (0, none; 1, few balloon cells; 2, many cells/prominent ballooning), and fibrosis stage (from 0, none to 4, cirrhosis). Visceral adipose tissue samples were also obtained from 18 patients and frozen until analyzed. All subjects gave their informed written consent to participate in this research study according to French legislation regarding Ethic and Human Research (Huriet-Serusclat law). The “Comité Consultatif de Protection des Personnes dans la Recherche Biomédicale de Nice” approved this study (07/04:2003, N° 03.017). *Control subjects*: Total RNA from 6 control livers was purchased from Stratagene (La Jolla, CA), Clontech (Mountain View, CA) and Biochain (Hayward, CA). The Stratagene and Biochain companies confirmed that histological findings were completely normal with no evidence of fatty liver disease. No clinical or biological data were available for these individuals but the absence of inflammatory process was corroborated by low CRP mRNA expression levels, as previously reported [10], [11]. Control subcutaneous adipose tissue was obtained from 4 lean subjects (2 females and 2 males; age, 37±11 years; BMI, 22.1±0.7 kg/m^2^) undergoing lipectomy for cosmetic purpose.

Sera from 9 lean patients and 70 morbidly obese patients (15 S0 patients, 23 S3 patients and 23 NASH patients with severe steatosis) were used to evaluate the circulating levels of IL6, TNFα, IP10 and MCP1 (realized by the IFR150, Toulouse, France) using the Milliplex Map from Millipore and endotoxin levels (LAL QCL-1000, Lonza). The abundances of palmitate as expressed relative to their values in a commercial serum sample (Sigma Aldrich, Ref. H4522) were evaluated in 25 morbidly obese patients using liquid chromatography-mass spectrometry (LC/MS).

### Real-time quantitative PCR analysis

Total RNA was extracted from human tissues using the RNable total RNA extraction kit (Eurobio, France) for liver biopsy and RNeasy Mini Kit (Qiagen, Contraboeuf, France) for adipose tissue. The samples were treated by Turbo DNA-free (Applied Biosystems, Contraboeuf, France) following the manufacturer's protocols. The quantity and quality of the isolated RNA were determined using the Agilent 2100 Bioanalyser with RNA 6000 Nano Kit (Agilent Technologies). Total RNA (1 µg) was reverse-transcribed with High Capacity cDNA Reverse Transcription Kit (Applied Biosystems, Foster City, CA). Real-time quantitative PCR was performed using the ABI PRISM 7900/7500 Fast Real Time PCR System and FAM dyes (Applied Biosystems, Contraboeuf, France) following the manufacturer's protocols in our Genomics facilities. The TaqMan gene expression assays were purchased from Applied Biosystems (Table S1). Gene expression values were normalized to the value of the housekeeping gene *RPLP0* (Ribosomal Phosphoprotein Large P0) and calculated based on the comparative cycle threshold Ct method (2^-ΔΔCt^).

### Statistical analysis

Statistical significance of differential gene expression between two study groups was determined using the non-parametric Kruskal-Wallis test with the ΔCt of each group. *P*<0.05 was considered as significant. However, the genes with either less than 1.5 fold of difference between two groups or a difference due to one patient in one group were no further considered as significantly modified.

## Supporting Information

Table S1 Genes included in this study
(0.10 MB PDF)

Table S2 Differentially expressed genes in visceral adipose tissue of NASH patients versus S0 and S3 patients
(0.08 MB PDF)
