# Supplementary material for: Hepatic Expression Patterns of Inflammatory and Immune Response Genes Associated with Obesity and NASH in Morbidly Obese Patients
Source: PLoS One. 2010 Oct 22;5(10):e13577. doi: 10.1371/journal.pone.0013577 (PMC2962651; doi:10.1371/journal.pone.0013577)
Supplement: Table S1 — Genes included in this study (0.10 MB PDF) [file pone.0013577.s001.pdf]

Table S1. Genes included in the study

Gene symbol	Gene alias	Gene name	NCBI number	Assay ID	Detected
**CD**					
CD3E		CD3E antigen, epsilon polypeptide (TiT3 complex)	NM_000733.2	Hs00167894_m1	Yes
CD4		CD4 antigen (p55)	NM_000616.2	Hs00181217_m1	Yes
CD8A		CD8 antigen, alpha polypeptide (p32)	NM_171827.1,NM_001768.4	Hs00233520_m1	Yes
CD11b	ITGAM, CR3A	CD11b antigen (p170); integrin, alpha M; complement component receptor 3, alpha (macrophage antigen alpha polypeptide)	NM_000632.2	Hs00355885_m1	Yes
CD18	ITGB2, LFA-1	CD18 antigen (p95); integrin, beta 2; lymphocyte function-associated antigen 1 (macrophage antigen 1 (mac-1) beta subunit)	NM_000211.1	Hs00164957_m1	Yes
CD19		CD19 antigen	NM_001770.3	Hs00174333_m1	Yes
CD28		CD28 antigen (Tp44)	NM_006139.1	Hs00174796_m1	Yes
CD34		CD34 antigen	NM_001025109.1,NM_001773.1	Hs00156373_m1	Yes
CD36		CD36 antigen (collagen type I receptor, thrombospondin receptor)	NM_001001547.1,NM_001001548.1,NM_000072.2	Hs00169627_m1	Yes
CD38		CD38 antigen (p45)	NM_001775.2	Hs00233552_m1	Yes
CD40		CD40 antigen (TNF receptor superfamily member 5)	NM_152854.1,NM_001250.3	Hs00386848_m1	Yes
CD44		CD44 antigen	NM_001001389.1,NM_001001390.1,NM_001001391.1,NM_001001392.1,NM_000610.3	Hs00153304_m1	Yes
CD47		CD47 antigen (Rh-related antigen, integrin-associated signal transducer)	NM_198793.2,NM_001025079.1,NM_001025080.1,NM_001777.3	Hs00179953_m1	Yes
CD48	LFA-3	CD48 antigen; lymphocyte function-associated antigen 3	NM_001778.2	Hs00381156_m1	Yes
CD54	ICAM1	CD54 antigen; intercellular adhesion molecule 1	NM_000201.1	Hs00164932_m1	Yes
CD62E	SELE	CD62E antigen; selectin E (endothelial adhesion molecule 1)	NM_000450.1	Hs00174057_m1	Yes
CD62P	SELP	CD62P antigen; selectin P (granule membrane protein 140kDa)	NM_003005.2	Hs00174583_m1	Yes
CD68	macrosialin	CD68 antigen	NM_001251.1	Hs00154355_m1	Yes
CD69	EA-1	CD69 antigen (p60); early T-cell activation antigen	NM_001781.1	Hs00156399_m1	Yes
CD80	B7-1	CD80 antigen (CD28 antigen ligand 1); B7-1 antigen	NM_005191.2	Hs00175478_m1	Yes
CD81	TAPA-1	CD81 antigen; target of antiproliferative antibody 1	NM_004356.3	Hs00174717_m1	Yes
CD86	B7-2	CD86 antigen (CD28 antigen ligand 2); B7-2 antigen	NM_175862.2,NM_006889.2	Hs00199349_m1	Yes
CD152	CTLA-4	CD152 antigen; cytotoxic T-lymphocyte-associated protein 4	NM_005214.2	Hs00175480_m1	Yes
CD154	CD40LG	CD40 ligand (TNF superfamily, member 5)	NM_000074.2	Hs00163934_m1	Yes
CD180		CD180 antigen	NM_005582.1	Hs00194403_m1	Yes
CD209		CD209 antigen	NM_021155.2	Hs00253550_m1	Yes
**Chemokines and chemokine receptors**					
CC chemokines and CC chemokine receptors					
CCL2	MCP-1	chemokine (C-C motif) ligand 2; monocyte chemoattractant protein 1	NM_002982.3	Hs00234140_m1	Yes
CCL3	MIP-1α	chemokine (C-C motif) ligand 3; macrophage inflammatory protein 1, alpha	NM_002983.1	Hs00234142_m1	Yes
CCL4,	MIP-1β	chemokine (C-C motif) ligand 4; macrophage inflammatory protein 1, beta	NM_002984.1	Hs00605740_g1	Yes
CCL5	RANTES	chemokine (C-C motif) ligand 5; regulated on activation, normal T cell expressed and secreted	NM_002985.2	Hs00174575_m1	Yes
CCL7	MCP-3	chemokine (C-C motif) ligand 7; monocyte chemoattractant protein 3	NM_006273.2	Hs00171147_m1	No
CCL19	MIP-3β	chemokine (C-C motif) ligand 19; macrophage inflammatory protein 3, beta	NM_006274.2	Hs00171149_m1	Yes
CCL21	6Ckine	chemokine (C-C motif) ligand 21	NM_002989.2	Hs00171076_m1	Yes
CCR2		chemokine (C-C motif) receptor 2	NM_000647.3	Hs00174150_m1	No
CCR4		chemokine (C-C motif) receptor 4	NM_005508.4	Hs99999919_m1	Yes
CCR5		chemokine (C-C motif) receptor 5	NM_000579.1	Hs00152917_m1	Yes
CCR7		chemokine (C-C motif) receptor 7	NM_001838.2	Hs00171054_m1	Yes
CXC chemokines and CXC chemokine receptors					
CXCL1	GROα	chemokine (C-X-C motif) ligand 1; growth-regulated oncogene, alpha	NM_001511.1	Hs00236937_m1	Yes
CXCL2	GROβ	chemokine (C-X-C motif) ligand 2; growth-regulated oncogene, beta	NM_002089.1	Hs00236966_m1	Yes
CXCL3	GROγ	chemokine (C-X-C motif) ligand 3; growth-regulated oncogene, gamma	NM_002090.2	Hs00171061_m1	Yes
CXCL8	IL8	chemokine (C-X-C motif) ligand 8; interleukin 8	NM_000584.2	Hs00174103_m1	Yes
CXCL9	Mig	chemokine (C-X-C motif) ligand 9; monokine induced by interferon-gamma	NM_002416.1	Hs00171065_m1	Yes
CXCL10	IP-10	chemokine (C-X-C motif) ligand 10; interferon-inducible protein 10	NM_001565.1	Hs00171042_m1	Yes
CXCL11	I-TAC	chemokine (C-X-C motif) ligand 11; interferon-inducible T cell alpha chemoattractant	NM_005409.3	Hs00171138_m1	Yes
CXCL16		chemokine (C-X-C motif) ligand 16	NM_022059.1	Hs00222859_m1	Yes
CXCR1	IL8RA	chemokine (C-X-C motif) receptor 1; interleukin 8 receptor, alpha	NM_000634.2	Hs00174146_m1	Yes
CXCR2	IL8RB	chemokine (C-X-C motif) receptor 2; interleukin 8 receptor, beta	NM_001557.2	Hs00174304_m1	Yes
CXCR3		chemokine (C-X-C motif) receptor 3	NM_001504.1	Hs00171041_m1	Yes
CXCR4		chemokine (C-X-C motif) receptor 4	NM_003467.2	Hs00237052_m1	Yes
**Semaphorins, plexins and neuropilins**					
SEMA3A		sema domain, immunoglobulin domain (Ig), short basic domain, secreted, (semaphorin) 3A	NM_006080.1	Hs00173810_m1	Yes
SEMA3B		sema domain, immunoglobulin domain (Ig), short basic domain, secreted, (semaphorin) 3B	NM_001005914.1,NM_004636.2	Hs00190328_m1	Yes
SEMA3C		sema domain, immunoglobulin domain (Ig), short basic domain, secreted, (semaphorin) 3C	NM_006379.2	Hs00170762_m1	Yes
SEMA3D		sema domain, immunoglobulin domain (Ig), short basic domain, secreted, (semaphorin) 3D	NM_152754.2	Hs00380877_m1	Yes
SEMA3E		sema domain, immunoglobulin domain (Ig), short basic domain, secreted, (semaphorin) 3E	NM_012431.1	Hs00180842_m1	Yes
SEMA3F		sema domain, immunoglobulin domain (Ig), short basic domain, secreted, (semaphorin) 3F	NM_004186.2	Hs00188273_m1	Yes
SEMA3G		sema domain, immunoglobulin domain (Ig), short basic domain, secreted, (semaphorin) 3G	NM_020163.1	Hs00220101_m1	Yes
SEMA4A		sema domain, immunoglobulin domain (Ig), transmembrane domain (TM) and short cytoplasmic domain, (semaphorin) 4A	NM_022367.2	Hs00223617_m1	Yes
SEMA4B		sema domain, immunoglobulin domain (Ig), transmembrane domain (TM) and short cytoplasmic domain, (semaphorin) 4B	NM_198925.1,NM_020210.2	Hs00384240_m1	Yes
SEMA4C		sema domain, immunoglobulin domain (Ig), transmembrane domain (TM) and short cytoplasmic domain, (semaphorin) 4C	NM_017789.3	Hs00215035_m1	Yes
SEMA4D		sema domain, immunoglobulin domain (Ig), transmembrane domain (TM) and short cytoplasmic domain, (semaphorin) 4D	NM_006378.2	Hs00174819_m1	Yes
SEMA4F		sema domain, immunoglobulin domain (Ig), transmembrane domain (TM) and short cytoplasmic domain, (semaphorin) 4F	NM_004263.2	Hs00188642_m1	Yes
SEMA4G		sema domain, immunoglobulin domain (Ig), transmembrane domain (TM) and short cytoplasmic domain, (semaphorin) 4G	NM_017893.2	Hs00706622_s1	Yes
SEMA5A		sema domain, seven thrombospondin repeats (type 1 and type 1-like), transmembrane domain (TM) and short cytoplasmic domain, (semaphorin) 5A	NM_003966.1	Hs00187651_m1	Yes
SEMA5B		sema domain, seven thrombospondin repeats (type 1 and type 1-like), transmembrane domain (TM) and short cytoplasmic domain, (semaphorin) 5B	NM_018987.1,NM_001031702.1	Hs00400720_m1	Yes
SEMA6A		sema domain, transmembrane domain (TM), and cytoplasmic domain, (semaphorin) 6A	NM_020796.2	Hs00221174_m1	Yes
SEMA6B		sema domain, transmembrane domain (TM), and cytoplasmic domain, (semaphorin) 6B	NM_032108.2,NM_133327.1	Hs00259527_m1	Yes
SEMA6C		sema domain, transmembrane domain (TM), and cytoplasmic domain, (semaphorin) 6C	NM_030913.3	Hs00229454_m1	Yes

SEMA6D		sema domain, transmembrane domain (TM), and cytoplasmic domain, (semaphorin) 6D	NM_024966.2,NM_020858.1,NM_153616.1,NM_153617.1,NM_153618.1,NM_153619.1	Hs00227965_m1	Yes
SEMA7A		sema domain, immunoglobulin domain (Ig), and GPI membrane anchor, (semaphorin) 7A	NM_003612.1	Hs00175305_m1	Yes
PLXNA1		plexin A1	NM_032242.2	Hs00413698_m1	Yes
PLXNA2		plexin A2	NM_025179.2	Hs00390230_m1	Yes
PLXNA3		plexin A3	NM_017514.2	Hs00250178_m1	Yes
PLXNA4B		plexin A4, B	NM_181775.2	Hs00297356_m1	Yes
PLXNB1		plexin B1	NM_002673.3	Hs00182227_m1	Yes
PLXNB3		plexin B3	NM_005393.1	Hs00193613_m1	Yes
PLXNC1		plexin C1	NM_005761.1	Hs00194968_m1	Yes
PLXND1		plexin D1	NM_015103.1	Hs00391129_m1	Yes
NRP1		neuropilin 1	NM_003873.5	Hs00818574_m1	Yes
NRP2		neuropilin 2	NM_018534.3,NM_201264.1,NM_201266.1,NM_201267.1,NM_201279.1,NM_003872.2	Hs00187290_m1	Yes
**Interleukin pathway**					
IL1A		interleukin 1, alpha	NM_000575.3	Hs00174092_m1	No
IL1B		interleukin 1, beta	NM_000576.2	Hs00174097_m1	Yes
IL1F9		interleukin 1 family, member 9	NM_019618.2	Hs00219742_m1	No
IL1R1		interleukin 1 receptor, type I	NM_000877.2	Hs00168392_m1	Yes
IL1R2		interleukin 1 receptor, type II	NM_173343.1,NM_004633.3	Hs00174759_m1	Yes
IL1RAP		interleukin 1 receptor accessory protein	NM_002182.2	Hs00158057_m1	Yes
IL1RN		interleukin 1 receptor antagonist	NM_173841.1,NM_173842.1,NM_173843.1,NM_000577.3	Hs00277299_m1	Yes
IL2		interleukin 2	NM_000586.2	Hs00174114_m1	No
IL2RA		interleukin 2 receptor, alpha	NM_000417.1	Hs00166229_m1	Yes
IL2RG		interleukin 2 receptor, gamma (severe combined immunodeficiency)	NM_000206.1	Hs00173950_m1	Yes
IL3		interleukin 3 (colony-stimulating factor, multiple)	NM_000588.3	Hs00174117_m1	No
IL3RA		interleukin 3 receptor, alpha (low affinity)	NM_002183.2	Hs00174356_m1	Yes
IL4		interleukin 4	NM_172348.1,NM_000589.2	Hs00174122_m1	No
IL4R		interleukin 4 receptor	NM_001008699.1,NM_000418.2	Hs00166237_m1	Yes
IL5		interleukin 5 (colony-stimulating factor, eosinophil)	NM_000879.2	Hs00174200_m1	No
IL6		interleukin 6 (interferon, beta 2)	NM_000600.1	Hs00174131_m1	Yes
IL6R		interleukin 6 receptor	NM_181359.1,NM_000565.2	Hs00794121_m1	Yes
IL6ST		interleukin 6 signal transducer (gp130 oncostatin M receptor)	NM_175767.1,NM_002184.2	Hs00174360_m1	Yes
IL7		interleukin 7	NM_000880.2	Hs00174202_m1	Yes
IL7R		interleukin 7 receptor	NM_002185.2	Hs00233682_m1	Yes
IL9		interleukin 9	NM_000590.1	Hs00174125_m1	No
IL9R		interleukin 9 receptor	NM_002186.2	Hs00174371_m1	No
IL10		interleukin 10	NM_000572.2	Hs00174086_m1	Yes
IL10RA		interleukin 10 receptor, alpha	NM_001558.2	Hs00387004_m1	Yes
IL10RB		interleukin 10 receptor, beta	NM_000628.3	Hs00175123_m1	Yes
IL11		interleukin 11	NM_000641.2	Hs00174148_m1	No
IL12A		interleukin 12A (p35) (natural killer cell stimulatory factor 1, cytotoxic lymphocyte maturation factor 1)	NM_000882.2	Hs00168405_m1	Yes
IL12B		interleukin 12B (p40) (natural killer cell stimulatory factor 2, cytotoxic lymphocyte maturation factor 2)	NM_002187.2	Hs00233688_m1	No
IL12RB1		interleukin 12 receptor, beta 1	NM_153701.1,NM_005535.1	Hs00538167_m1	Yes
IL12RB2		interleukin 12 receptor, beta 2	NM_001559.2	Hs00155486_m1	Yes
IL13		interleukin 13	NM_002188.2	Hs00174379_m1	No
IL13RA2		interleukin 13 receptor, alpha 2	NM_000640.2	Hs00152924_m1	Yes
IL15		interleukin 15	NM_000585.2	Hs00174106_m1	Yes
IL15RA		interleukin 15 receptor, alpha	NM_002189.2	Hs00233692_m1	Yes
IL16	LCF	interleukin 16, lymphocyte chemoattractant factor	NM_172217.1,NM_004513.3	Hs00189606_m1	Yes
IL17	CTLA-8	interleukin 17, cytotoxic T-lymphocyte-associated serine esterase 8	NM_002190.2	Hs00174383_m1	No
IL17B		interleukin 17B	NM_014443.2	Hs00205368_m1	No
IL17D		interleukin 17D	NM_138284.1	Hs00370528_m1	No
IL17R		interleukin 17 receptor	NM_014339.3	Hs00234888_m1	Yes
IL18	IGIF	interleukin 18, interferon-gamma-inducing factor	NM_001562.2	Hs00155517_m1	Yes
IL18BP		interleukin 18 binding protein	NM_173042.1,NM_173044.1,NM_005699.2	Hs00271720_m1	Yes
IL18R1		interleukin 18 receptor 1	NM_003855.2	Hs00175381_m1	Yes
IL18RAP		interleukin 18 receptor accessory protein	NM_003853.2	Hs00187256_m1	Yes
IL20		interleukin 20	NM_018724.3	Hs00218888_m1	No
IL22		interleukin 22	NM_020525.4	Hs00220924_m1	No
IL22RA1		interleukin 22 receptor, alpha 1	NM_021258.2	Hs00222035_m1	Yes
IL27RA		interleukin 27 receptor, alpha	NM_004843.2	Hs00175472_m1	Yes
NFIL3		nuclear factor, interleukin 3 regulated	NM_005384.2	Hs00705412_s1	Yes
**JAK-STAT-SOCS pathway**					
JAK1		Janus kinase 1 (a protein tyrosine kinase)	NM_002227.1	Hs00233820_m1	Yes
JAK2		Janus kinase 2 (a protein tyrosine kinase)	NM_004972.2	Hs00234567_m1	Yes
JAK3		Janus kinase 3 (a protein tyrosine kinase, leukocyte)	NM_000215.2	Hs00169663_m1	Yes
STAT1		signal transducer and activator of transcription 1, 91kDa	NM_139266.1,NM_007315.2	Hs00234829_m1	Yes
STAT2		signal transducer and activator of transcription 2, 113kDa		Hs00237139_m1	Yes
STAT3		signal transducer and activator of transcription 3 (acute-phase response factor)	NM_139276.2	Hs00234174_m1	Yes
STAT4		signal transducer and activator of transcription 4	NM_003151.2	Hs00231372_m1	Yes
SOCS1		suppressor of cytokine signaling 1	NM_003745.1	Hs00705164_s1	Yes
SOCS2		suppressor of cytokine signaling 2	NM_003877.3	Hs00919620_m1	Yes
SOCS3		suppressor of cytokine signaling 3	NM_003955.3	Hs00269575_s1	Yes
SOCS4		suppressor of cytokine signaling 4	NM_199421.1,NM_080867.2	Hs00365582_s1	Yes
SOCS5		suppressor of cytokine signaling 5	NM_014011.4	Hs00367107_m1	Yes
SOCS6		suppressor of cytokine signaling 6	NM_004232.2	Hs00377781_m1	Yes

SOCS7		suppressor of cytokine signaling 7	NM_014598.1	Hs00389987_m1	Yes
**IFN pathway**					
IFNA2		interferon, alpha 2	NM_000605.2	Hs00265051_s1	No
IFNA8		interferon, alpha 8	NM_002170.2	Hs00266883_s1	No
IFNA21		interferon, alpha 21	NM_002175.1	Hs00356648_s1	No
IFNAR1		interferon (alpha, beta and omega) receptor 1	NM_000629.2	Hs00265057_m1	Yes
IFNAR2		interferon (alpha, beta and omega) receptor 2	NM_207584.1,NM_207585.1,NM_000874.3	Hs00174198_m1	Yes
IFNB1		interferon, beta 1, fibroblast	NM_002176.2	Hs00277188_s1	No
IFNG		interferon, gamma	NM_000619.2	Hs00174143_m1	Yes
IFNGR1		interferon gamma receptor 1	NM_000416.1	Hs00166223_m1	Yes
IRF1		interferon regulatory factor 1	NM_002198.1	Hs00233698_m1	Yes
IRF2		interferon regulatory factor 2	NM_002199.2	Hs00180006_m1	Yes
IRF3		interferon regulatory factor 3	NM_001571.2	Hs00155574_m1	Yes
IRF4		interferon regulatory factor 4	NM_002460.1	Hs00180031_m1	Yes
IRF5		interferon regulatory factor 5	NM_032643.3,NM_002200.3	Hs00158114_m1	Yes
IRF6		interferon regulatory factor 6	NM_006147.2	Hs00196213_m1	Yes
IRF7		interferon regulatory factor 7	NM_001572.2,NM_004029.1,NM_004031.1	Hs00185375_m1	Yes
IFI16		interferon, gamma-inducible protein 16	NM_005531.1	Hs00194261_m1	Yes
G1P3		interferon, alpha-inducible protein (clone IFI-6-16)	NM_022872.1,NM_022873.1,NM_002038.2	Hs00242571_m1	Yes
**TNFα and TGFβ pathways**					
TNF		tumor necrosis factor (TNF superfamily, member 2)	NM_000594.2	Hs00174128_m1	Yes
LTA		lymphotoxin alpha (TNF superfamily, member 1)	NM_000595.2	Hs00236874_m1	Yes
LTB		lymphotoxin beta (TNF superfamily, member 3)	NM_009588.1,NM_002341.1	Hs00242739_m1	Yes
TNFRSF14		tumor necrosis factor receptor superfamily, member 14 (herpesvirus entry mediator)	NM_003820.2	Hs00187058_m1	Yes
TNFRSF18		tumor necrosis factor receptor superfamily, member 18	NM_148901.1,NM_148902.1,NM_004195.2	Hs00188346_m1	No
TNFRSF1A		tumor necrosis factor receptor superfamily, member 1A	NM_001065.2	Hs00533560_m1	Yes
TNFRSF1B		tumor necrosis factor receptor superfamily, member 1B	NM_001066.2	Hs00153550_m1	Yes
TRAF1		TNF receptor-associated factor 1	NM_005658.3	Hs00194639_m1	Yes
TRAF2		TNF receptor-associated factor 2		Hs00377454_m1	No
TRAF3		TNF receptor-associated factor 3	NM_145726.1,NM_145725.1,NM_003300.2	Hs00377462_m1	Yes
TRAF3IP1		TNF receptor-associated factor 3 interacting protein 1	NM_015650.2	Hs00249015_m1	Yes
TRAF4		TNF receptor-associated factor 4	NM_145751.1,NM_004295.2	Hs00188755_m1	Yes
TRAF5		TNF receptor-associated factor 5	NM_145759.1,NM_004619.2	Hs00182979_m1	Yes
TRAF6		TNF receptor-associated factor 6	NM_004620.2	Hs00371508_m1	Yes
TRAF7		TNF receptor-associated factor 7	NM_032271.2,NM_206835.1	Hs00260228_m1	Yes
TRAP1		TNF receptor-associated protein 1	NM_016292.1	Hs00212476_m1	Yes
TTRAP		TRAF and TNF receptor associated protein	NM_016614.2	Hs00213282_m1	Yes
TGFB1		transforming growth factor, beta 1	NM_000660.3	Hs00171257_m1	Yes
TGFBR1		transforming growth factor, beta receptor I (activin A receptor type II-like kinase, 53kDa)	NM_004612.2	Hs00610319_m1	Yes
TGFBR2		transforming growth factor, beta receptor II (70/80kDa)	NM_003242.4,NM_001024847.1	Hs00559661_m1	Yes
**NFκB pathway**					
IKBKB		inhibitor of kappa light polypeptide gene enhancer in B-cells, kinase beta	NM_001556.1	Hs00826074_m1	Yes
IKBKG		inhibitor of kappa light polypeptide gene enhancer in B-cells, kinase gamma	NM_003639.2	Hs00416633_m1	No
NFKB1		nuclear factor of kappa light polypeptide gene enhancer in B-cells 1 (p105)	NM_003998.2	Hs00765730_m1	Yes
NFKB2		nuclear factor of kappa light polypeptide gene enhancer in B-cells 2 (p49/p100)	NM_002502.2	Hs00174517_m1	Yes
NFKBIA		nuclear factor of kappa light polypeptide gene enhancer in B-cells inhibitor, alpha	NM_020529.1	Hs00153283_m1	Yes
NFKBIB		nuclear factor of kappa light polypeptide gene enhancer in B-cells inhibitor, beta	NM_001001716.1,NM_002503.3	Hs00182115_m1	Yes
REL		v-rel reticuloendotheliosis viral oncogene homolog (avian)	NM_002908.2	Hs00231279_m1	Yes
RELA		v-rel reticuloendotheliosis viral oncogene homolog A, nuclear factor of kappa light polypeptide gene enhancer in B-cells 3, p65 (avian)	NM_021975.2	Hs00153294_m1	Yes
RELB		v-rel reticuloendotheliosis viral oncogene homolog B, nuclear factor of kappa light polypeptide gene enhancer in B-cells 3 (avian)	NM_006509.2	Hs00232399_m1	Yes
**c-Jun N-terminal kinases**					
MAPK8	JNK1	mitogen-activated protein kinase 8; c-Jun N-terminal kinase 1	NM_139046.1,NM_139047.1,NM_139049.1,NM_002750.2	Hs00177083_m1	Yes
MAPK9	JNK2	mitogen-activated protein kinase 9; c-Jun N-terminal kinase 2	NM_139068.1,NM_139069.1,NM_139070.1,NM_002752.3	Hs00177102_m1	Yes
**Toll-like receptors and lipopolysaccharide signaling pathway**					
TLR2		toll-like receptor 2	NM_003264.3	Hs00610101_m1	Yes
TLR4		toll-like receptor 4	NM_138554.1,NM_003266.2	Hs00152939_m1	Yes
TLR6		toll-like receptor 6	NM_006068.2	Hs00271977_s1	Yes
CD14		CD14 antigen (lipopolysaccharide receptor)	NM_000591.1	Hs00169122_g1	Yes
LBP		lipopolysaccharide binding protein	NM_004139.2	Hs00188074_m1	Yes
LITAF		lipopolysaccharide-induced TNF factor	NM_004862.2	Hs00191583_m1	Yes
CHUK	IKKα	conserved helix-loop-helix ubiquitous kinase; Ikappa B kinase, alpha	NM_001278.3	Hs00175141_m1	Yes
**Matrix proteases and inhibitors of matrix proteases**					
ADAM10		ADAM metallopeptidase domain 10	NM_001110.1	Hs00153853_m1	Yes
MMP1		matrix metallopeptidase 1 (interstitial collagenase)	NM_002421.2	Hs00233958_m1	No
MMP2		matrix metallopeptidase 2 (gelatinase A, 72kDa gelatinase, 72kDa type IV collagenase)	NM_004530.1	Hs00234422_m1	Yes
MMP3		matrix metallopeptidase 3 (stromelysin 1, progelatinase)	NM_002422.2	Hs00233962_m1	No
MMP8		matrix metallopeptidase 8 (neutrophil collagenase)	NM_002424.1	Hs00233972_m1	Yes
MMP9		matrix metallopeptidase 9 (gelatinase B, 92kDa gelatinase, 92kDa type IV collagenase)	NM_004994.1	Hs00234579_m1	Yes
MMP10		matrix metallopeptidase 10 (stromelysin 2)	NM_002425.1	Hs00233987_m1	No
MMP11		matrix metallopeptidase 11 (stromelysin 3)	NM_005940.3	Hs00171829_m1	Yes
MMP13		matrix metallopeptidase 13 (collagenase 3)	NM_002427.2	Hs00233992_m1	No
MMP14		matrix metallopeptidase 14 (membrane-inserted)	NM_004995.2	Hs00237119_m1	Yes
MMP15		matrix metallopeptidase 15 (membrane-inserted)	NM_002428.2	Hs00233997_m1	Yes
PLAT	tPA	plasminogen activator, tissue	NM_033011.1,NM_000930.2,NM_000931.2	Hs00263492_m1	Yes
PLAU	uPA	plasminogen activator, urokinase	NM_002658.2	Hs00170182_m1	Yes

SERPIN1	PAI-1	serpin peptidase inhibitor, clade E (nexin, plasminogen activator inhibitor type 1), member 1	NM_000602.1	Hs00167155_m1	Yes
TIMP1		TIMP metallopeptidase inhibitor 1	NM_003254.1	Hs00171558_m1	Yes
TIMP2		TIMP metallopeptidase inhibitor 2	NM_003255.3	Hs00234278_m1	Yes
TIMP3		TIMP metallopeptidase inhibitor 3 (Sorsby fundus dystrophy, pseudoinflammatory)	NM_000362.3	Hs00165949_m1	Yes
**Cell junction**					
GJA1		gap junction protein, alpha 1, 43kDa (connexin 43)	NM_000165.2	Hs00748445_s1	Yes
GJA3		gap junction protein, alpha 3, 46kDa (connexin 46)	NM_021954.2	Hs00254296_s1	No
GJA12		gap junction protein, alpha 12, 47kDa	NM_020435.2	Hs00252713_s1	Yes
GJB1		gap junction protein, beta 1, 32kDa (connexin 32, Charcot-Marie-Tooth neuropathy, X-linked)	NM_000166.2	Hs00702141_s1	Yes
GJB2		gap junction protein, beta 2, 26kDa (connexin 26)	NM_004004.3	Hs00269615_s1	Yes
CLDN5		claudin 5 (transmembrane protein deleted in velocardiofacial syndrome)	NM_003277.2	Hs00533949_s1	Yes
CLDN10		claudin 10	NM_006984.3	Hs00199599_m1	Yes
CLDN11		claudin 11 (oligodendrocyte transmembrane protein)	NM_005602.4	Hs00194440_m1	Yes
**Acute phase protein**					
CRP		C-reactive protein, pentraxin-related	NM_000567.2,X56692.1,X56214.1,BC020766.1	Hs00357041_m1	Yes
**Housekeeping gene**					
RPLP0		ribosomal protein, large, P0	NM_053275.3,NM_001002.3	Hs99999902_m1	Yes
