# Supplementary material for: Hepatic Expression Patterns of Inflammatory and Immune Response Genes Associated with Obesity and NASH in Morbidly Obese Patients
Source: PLoS One. 2010 Oct 22;5(10):e13577. doi: 10.1371/journal.pone.0013577 (PMC2962651; doi:10.1371/journal.pone.0013577)
Supplement: Table S2 — Differentially expressed genes in visceral adipose tissue of NASH patients versus S0 and S3 patients (0.08 MB PDF) [file pone.0013577.s002.pdf]

Table S2. Differentially expressed genes in visceral adipose tissue of NASH patients versus S0 and S3 patients

Gene symbol	S0	S3	NASH
CD			
CD18	1.00±0.15	1.78±0.26*	1.80±0.18*
CD80	1.00±0.20	1.25±0.23	2.49±0.50*/§
Chemokines			
CXCL11	1.00±0.10	2.77±0.63*	3.51±0.78*
CXCL16	1.00±0.06	1.63±0.19*	1.71±0.20*
CXCL9	1.00±0.04	1.77±0.44	3.40±0.94*/§
Interleukin pathway			
IL27RA	1.00±0.10	1.79±0.43*	1.38±0.10*
IL3RA	1.00±0.11	1.76±0.26*	1.60±0.20*
IL15	1.00±0.07	1.59±0.10*	1.49±0.14*
IL12RB1	1.00±0.12	1.77±0.26*	1.60±0.23*
IL18BP	1.00±0.05	1.46±0.22*	1.65±0.12*

S0: patients with normal liver histology (n=6), S3: patients with severe steatosis (n=6), NASH: patients with severe steatosis and NASH (n=6). Results are expressed relative to S0 patients (mean±SEM) and were compared by using the non parametric Kruskal-Wallis test. *P<0.05 compared with S0 patients and §P<0.05 compared with S3 patients.
